# Risk Factors for Proximal Junctional Kyphosis in Osteoporotic Versus Non-Osteoporotic Patients After Adult Spinal Deformity Surgery

**DOI:** 10.3390/jcm15166479

**Published:** 2026-08-21

**Authors:** Tae Soo Shin, Jin-Sung Park, Dong-Ho Kang, Jun-Seok Oh, Se-Jun Park

**Affiliations:** Department of Orthopedic Surgery, Samsung Medical Center, School of Medicine, Sungkyunkwan University, Seoul 06351, Republic of Korea

**Keywords:** osteoporosis, adult spinal deformity, proximal junctional kyphosis, risk factors

## Abstract

**Background/Objectives**: Proximal junctional kyphosis (PJK) is a frequent mechanical complication following adult spinal deformity (ASD) surgery, and osteoporosis is a well-recognized risk factor. However, whether the underlying risk factor profiles for PJK differ according to bone mineral density status remains unclear. This study aimed to identify and compare independent risk factors for PJK between osteoporotic and non-osteoporotic patients following long-segment posterior spinal fusion for ASD. **Methods**: This retrospective cohort study included 356 patients who underwent ≥5-level posterior fusion to the sacrum or pelvis for ASD with a 2-year follow-up. Patients were stratified into a non-osteoporotic (non-OP; *n* = 284) and an osteoporotic (OP; *n* = 72) group based on preoperative dual-energy X-ray absorptiometry (T-score ≤ −2.5). PJK was defined as a proximal junctional angle (PJA) ≥20° with an increase of ≥10° from the preoperative value. Demographic, surgical, and radiographic variables were analyzed separately for each group using univariate and stepwise multivariate logistic regression analyses. Predictive performance was assessed using receiver operating characteristic curve analysis. **Results**: The overall PJK incidence tended to be higher in the OP group than the non-OP group (36.1% vs. 24.6%, *p* = 0.055), and fracture-type PJK occurred significantly more frequently in osteoporotic patients (29.2% vs. 15.8%, *p* = 0.016). The variables that reached statistical significance differed between the two groups. In the non-OP group, lower Hounsfield units at the upper instrumented vertebra (UIV; OR = 0.990, *p* = 0.011), higher preoperative PJA (OR = 1.098, *p* < 0.001), and greater L1 pelvic angle (L1PA) offset indicating relative overcorrection (OR = 1.136, *p* < 0.001) were independent predictors of PJK. In the OP group, higher preoperative PJA (OR = 1.149, *p* = 0.007) and greater age-adjusted pelvic incidence–lumbar lordosis offset (OR = 1.052, *p* = 0.039) were identified as independent risk factors. Multivariable models demonstrated acceptable discriminative ability in both groups (area under the curve [AUC] = 0.741, 95% confidence interval [CI] = 0.671–0.804 for the non-OP group; AUC = 0.753, 95% CI = 0.632–0.867 for the OP group; optimism-corrected AUC 0.729 and 0.735 after bootstrap internal validation). **Conclusions:** The variables associated with PJK differed according to osteoporosis status following ASD surgery. Careful UIV selection avoiding kyphotic junctional alignment may be relevant for both groups. Avoiding overcorrection relative to the age-adjusted target may warrant particular attention in osteoporotic patients, while UIV bone quality and L1PA overcorrection may be relevant to surgical planning in non-osteoporotic patients.

## 1. Introduction

Adult spinal deformity (ASD) encompasses a spectrum of sagittal and coronal spinal malalignment that significantly impacts quality of life, particularly affecting the aging population. With increasing life expectancy, the number of patients with osteoporosis is steadily rising, imposing a clinical challenge in treating these patients [1,2]. A recent systematic review of literature involving patients over the age of 50 undergoing spinal surgery found the prevalence of osteoporosis to be 34.2% [1].

Proximal junctional kyphosis (PJK) remains a challenging mechanical complication following ASD surgery, with reported incidence rates ranging from 20% to 40% [3,4]. Among the multifactorial etiologies, prior studies have commonly suggested that osteoporosis independently increases the risk of PJK development [5,6,7,8,9,10,11,12]. Osteoporosis may compromise bone quality at the uppermost instrumented vertebra, predisposing patients to vertebral fracture and fixation failure. In addition, considering that these patients are typically elderly, not only bone quality but also the condition of surrounding soft tissue, including paraspinal muscle and ligamentous structure, is likely to be deteriorated, which may collectively increase the risk of PJK. These considerations suggest that the contributing risk factors for PJK in osteoporotic patients may differ from those observed in patients without osteoporosis. We hypothesized that distinct risk factor profiles would emerge between patients with and without osteoporosis.

The primary objective of this study was to identify and compare risk factors for PJK development between patients with and without osteoporosis following long-segment posterior spinal fusion for ASD. Understanding these risk factors may enable surgeons to optimize patient selection and surgical planning, thereby reducing the burden of this common complication.

## 2. Materials and Methods

### 2.1. Study Design

This retrospective cohort study utilized a prospectively collected database from a tertiary referral center. The institutional review board approved this study and waived the requirement for informed consent due to its retrospective design (IRB number: 2026-06-095).

### 2.2. Study Population

The study included consecutive patients who underwent corrective surgery for symptomatic ASD between 2014 and 2023. Inclusion criteria were as follows: ASD defined by at least one of the following parameters—pelvic incidence minus lumbar lordosis (PI-LL) mismatch ≥10°, pelvic tilt (PT) ≥25°, sagittal vertical axis (SVA) ≥5 cm, or a coronal Cobb angle ≥20°; ≥5-level fusion to the sacrum or pelvis; and a follow-up period of 2 years. Radiographic follow-up was conducted at standardized intervals: 6 weeks, 3 months, 6 months, and biannually thereafter. Because a minimum 2-year follow-up was an inclusion criterion and radiographic observation was censored administratively at 24 months, every analysed patient contributed a full 24 months of follow-up and there was no loss to follow-up within the analysed cohort. The selection of the upper instrumented vertebra (UIV) was determined by the surgeon’s judgment considering the extent of deformity and preoperative flexibility. Pelvic fixation using conventional iliac screws was routinely performed in primary lumbosacral fusion, while fusion was terminated at the sacrum without pelvic fixation in patients with milder deformities and pre-existing lumbosacral solid fusion, either from prior surgery or due to a lumbosacral transitional vertebra. Exclusion criteria were: (1) non-degenerative aetiology of deformity; (2) follow-up shorter than 2 years; (3) reoperation during follow-up for an indication unrelated to junctional complications, such as surgical site infection or pseudarthrosis, after which the original construct was no longer intact; and (4) inadequate radiographic studies, defined as the absence of standing whole-spine radiographs at follow-up. Patients whose radiographs were adequate but in whom an individual parameter could not be measured were retained and handled as missing data. Osteoporosis was defined as a T-score ≤ −2.5 on dual-energy X-ray absorptiometry (DEXA) obtained within 3 months before surgery. Because bone mineral density measured at the lumbar spine can be falsely elevated by degenerative change and aortic calcification, the lumbar value was not used in isolation: the lowest T-score among the spine and hip regions, excluding Ward’s area, was used to define osteoporosis. Patients were stratified into two groups: non-osteoporosis (non-OP) (T-score > −2.5) and osteoporosis (OP) groups (T-score ≤ −2.5). The proximal junctional angle (PJA) was measured between the caudal endplate of the UIV and the cephalad endplate of UIV+2 on standing lateral radiographs, with kyphosis recorded as positive and lordosis as negative. PJK was defined as a PJA ≥20° with an increase of ≥10° from the preoperative value [13]. PJK was classified as fracture-type when a new compression or burst fracture of the UIV or UIV+1 was present, and as soft tissue failure-type when the junctional angular change occurred in the absence of any vertebral fracture. Fracture was identified by comparing the postoperative UIV and UIV+1 with the corresponding preoperative vertebrae on standing radiographs; when the radiographic appearance was equivocal, computed tomography was obtained to confirm or exclude fracture. Screw pullout or loosening at the UIV occurring without vertebral fracture was classified as soft tissue failure-type. Failure mode was adjudicated independently by two spine surgeons blinded to bone mineral density status, with disagreements resolved by consensus with a third senior author. Revision surgery for junctional complications during follow-up was recorded as a secondary clinical outcome. All procedures were performed by three senior spine surgeons at a single tertiary centre. Cement augmentation was applied at the UIV only and not at UIV+1. No ligament or tether augmentation was used, and no standardised proximal-junction prophylaxis protocol was in place during the study period.

### 2.3. Presumed Risk Factors

Potential risk factors were categorized into demographic, surgical, and radiographic domains. Demographic variables included age, sex, primary diagnosis (flatback deformity vs. kyphoscoliosis), American Society of Anesthesiologists (ASA) physical status classification, frailty status using the modified frailty index-5 (mFI-5) (classified as robust [mFI-5 = 0], pre-frail [mFI-5 = 1], and frail [mFI-5 ≥ 2]) [14], body mass index (BMI), T-score, Hounsfield units (HU), and preoperative use of parathyroid hormone (PTH) for at least 3 months. The agent used was teriparatide, administered at 20 μg daily by subcutaneous injection for at least 3 months before surgery. It was prescribed at the discretion of the treating surgeon when severe osteoporosis on DEXA was accompanied by a markedly osteopenic appearance of the lumbar spine on plain radiographs, and adherence was ascertained from patient report at outpatient follow-up. The exposure was defined by preoperative use; postoperative continuation was at the discretion of the treating physician and was not systematically recorded. Hounsfield units were measured on preoperative non-contrast computed tomography obtained as part of routine preoperative planning. CT examinations were acquired on several scanners over the 2014–2023 study period under routine clinical protocols, so acquisition parameters were not uniform across the cohort and are not retrievable for every patient. A single elliptical region of interest was placed within the cancellous portion of the UIV vertebral body on an axial image, excluding cortical bone, the posterior elements, and any focal sclerosis or vascular channel; values were not averaged across multiple slices. Surgical factors included prior lumbar fusion history, anterior lumbar interbody fusion (ALIF) at L5-S1, lateral lumbar interbody fusion (LLIF) at ≥L4-5, pedicle subtraction osteotomy (PSO), cement augmentation at UIV, use of a transverse process (TP) hook at UIV+1, UIV screw angle with positive values indicating a cephalad trajectory, number of rods, and total fusion length. Radiographic variables included preoperative and postoperative (6-week) sagittal parameters. All radiographic and CT parameters were measured by two independent observers who were blinded to bone mineral density status and to PJK outcome, and the averaged values were used for analysis. Before surgery, the following sagittal parameters were measured: pelvic incidence (PI), PI-LL, sacral slope (SS), PT, thoracic kyphosis (TK), T1 pelvic angle (T1PA), SVA, and proximal junctional angle (PJA). Postoperatively (at 6 weeks), positional parameters subject to passive changes—SS, PT, TK, T1PA, SVA, and PJA—were excluded from the analysis. Instead, variables known to affect PJK development were included, such as change in lumbar lordosis (LL) [15], PI-LL, age-adjusted PI-LL [16], and L1 pelvic angle (L1PA) [17]. L1PA was measured as the angle formed by a line from the center of the L1 vertebral body to the femoral head axis and a line from the femoral head axis to the center of the S1 endplate. Age-adjusted PI-LL offset = [(age − 55) ÷ 2 + 3] − measured postoperative (PI − LL). L1PA offset = (PI × 0.5 − 21) − measured postoperative L1PA. For both parameters the normative value is the minuend and the measured value the subtrahend, so that a positive offset denotes correction beyond the age-adjusted or PI-based target, that is, relative overcorrection, and a negative offset denotes residual under-correction. When early PJK was identified prior to the 6-week postoperative evaluation, radiographic parameters were measured on images acquired before discharge (approximately 2 weeks postoperatively).

### 2.4. Statistical Analysis

Continuous variables were expressed as means with standard deviations, and categorical variables as frequencies with percentages. The overall incidence of PJK was compared between the non-OP and OP groups using Fisher’s exact test. The distribution of PJK modes (fracture-type or soft tissue failure-type) was compared between the non-OP and OP groups using the chi-square test. Additionally, Fisher’s exact test was specifically performed to compare the incidence of fracture-type PJK between groups. Among patients who developed PJK, the timing of the event was summarised as the median time from surgery to the radiograph on which PJK was first documented.

For risk factor analysis, univariate analyses of demographic, surgical, and radiographic risk factors were performed using independent t-tests and chi-square tests for each OP group. Subsequently, stepwise multivariate logistic regression analysis was conducted to identify the independent predictors of PJK. To minimize the risk of model overfitting given the limited number of events, only variables with statistical significance (*p* < 0.05) in univariate analysis were included in the multivariate model. In addition to the stepwise model, a clinically prespecified model was fitted in each group. Eleven covariates were fixed in advance on clinical grounds and retained irrespective of their univariate *p* values: age, body mass index, HU at the UIV, previous lumbar fusion, UIV at T11-L1, pelvic fixation, cement augmentation at the UIV, transverse process hook at UIV+1, preoperative PJA, age-adjusted PI-LL offset and L1PA offset. Cement augmentation and the transverse process hook were included for adjustment only and are not interpreted as risk estimates, because both are applied preferentially to patients judged preoperatively to be at high risk. The model was fitted by Firth penalized logistic regression to correct small-sample bias in the osteoporotic subgroup (Supplementary Table S3). To evaluate the predictive performance of identified risk factors, receiver operating characteristic (ROC) curve analysis was performed separately for non-OP and OP groups. The area under the curve (AUC) with 95% confidence intervals (CI) was calculated, and optimal cutoff values were determined using Youden’s index. Finally, multivariable logistic regression models were constructed to assess the combined predictive performance of all risk factors within each group. Model performance was evaluated using sensitivity, specificity, positive predictive value (PPV), negative predictive value (NPV), and overall accuracy. Because radiographic parameters were measured on the before-discharge film in patients whose PJK was identified before the 6-week visit, a sensitivity analysis was performed after excluding those patients, so that every measurement in the remaining cohort came from the uniformly timed 6-week radiograph (Supplementary Table S4).

Whether the effect of each candidate predictor differed by osteoporosis status was tested formally rather than inferred from separate subgroup models. For every variable that reached significance on univariate analysis in either group, a logistic model containing osteoporosis status, the variable and their interaction was fitted in the whole cohort, and the ratio of odds ratios with its 95% confidence interval was taken as the measure of effect modification (Supplementary Table S1). The unadjusted comparison of PJK incidence between the osteoporosis groups was repeated with adjustment for age, sex, previous lumbar fusion, ALIF at L5-S1, LLIF at ≥L4-5, pelvic incidence and preoperative PTH use. Bone mineral density was additionally analysed as a graded exposure, using a three-category classification of normal, osteopenic and osteoporotic with a chi-square test for trend, and using the T-score as a continuous variable. The multivariable models were internally validated with 1000 bootstrap resamples using Harrell’s optimism-correction procedure, repeating the full variable-selection procedure within each resample; optimism-corrected discrimination, the calibration slope, calibration-in-the-large and the Brier score against the null model are reported in Supplementary Table S2, and the calibration curves in Supplementary Figure S1. Areas under the curve were compared using the DeLong test. A further sensitivity analysis refitted the non-osteoporotic model with revision surgery for junctional complications as the outcome, an endpoint that does not depend on any angular threshold.

Hounsfield units could not be measured in 17 patients (4.8%) who did not undergo preoperative computed tomography, and the preoperative proximal junctional angle could not be measured in 12 patients (3.4%) in whom the upper thoracic spine was not adequately visualised on the standing lateral radiograph. The UIV screw angle was unavailable in 12 patients (3.4%) and preoperative thoracic kyphosis in 118 (33.1%). These patients had adequate radiographic studies and were retained in the cohort; only patients without standing whole-spine radiographs at follow-up were excluded. Missing values were not imputed. All analyses were performed on complete cases, and the analytic sample is stated for each table. Statistical analyses were performed using Python version 3.10 (Python Software Foundation, Beaverton, OR, USA). A *p*-value of <0.05 was considered statistically significant.

## 3. Results

### 3.1. Study Population and Baseline Characteristics

Of 407 patients who underwent ≥5-level fusion to the sacrum or pelvis for ASD, 51 were excluded (15 for non-degenerative aetiology, 25 for follow-up shorter than 2 years, 9 for reoperation during follow-up for an indication unrelated to junctional complications, and 2 for inadequate radiographic studies), leaving 356 patients for analysis. Fixation extended to the pelvis in 258 patients (72.5%) and terminated at the sacrum in 98 (27.5%), with no difference between the groups (72.5% vs. 72.2%, *p* = 0.958). They were stratified into non-OP (N = 284; 79.8%) and OP (N = 72; 20.2%) groups based on the presence of osteoporosis. The OP group was significantly older (72.0 vs. 69.2 years, *p* = 0.002) and had a higher proportion of females (97.2% vs. 86.3%, *p* = 0.009) (Table 1). The OP group also had significantly lower T-scores (−3.2 vs. −1.0, *p* < 0.001) and lower HU at UIV (90.8 vs. 119.3, *p* < 0.001). Additionally, the OP group had higher rates of preoperative PTH use (20.8% vs. 9.9%, *p* = 0.011) but lower rates of prior lumbar fusion (31.9% vs. 45.4%, *p* = 0.039), ALIF at L5-S1 (31.9% vs. 46.1%, *p* = 0.030), and LLIF at ≥ L4-5 (54.2% vs. 70.4%, *p* = 0.009). Other demographic and surgical characteristics were comparable between groups. Among the preoperative sagittal parameters, pelvic incidence was higher in the OP group (56.4° vs. 53.0°, *p* = 0.017); the remaining preoperative sagittal parameters did not differ significantly between the groups. The patient flow, with the reason for each exclusion, is shown in Supplementary Figure S2.

### 3.2. PJK Incidence by Osteoporosis Status

**Figure 1:**
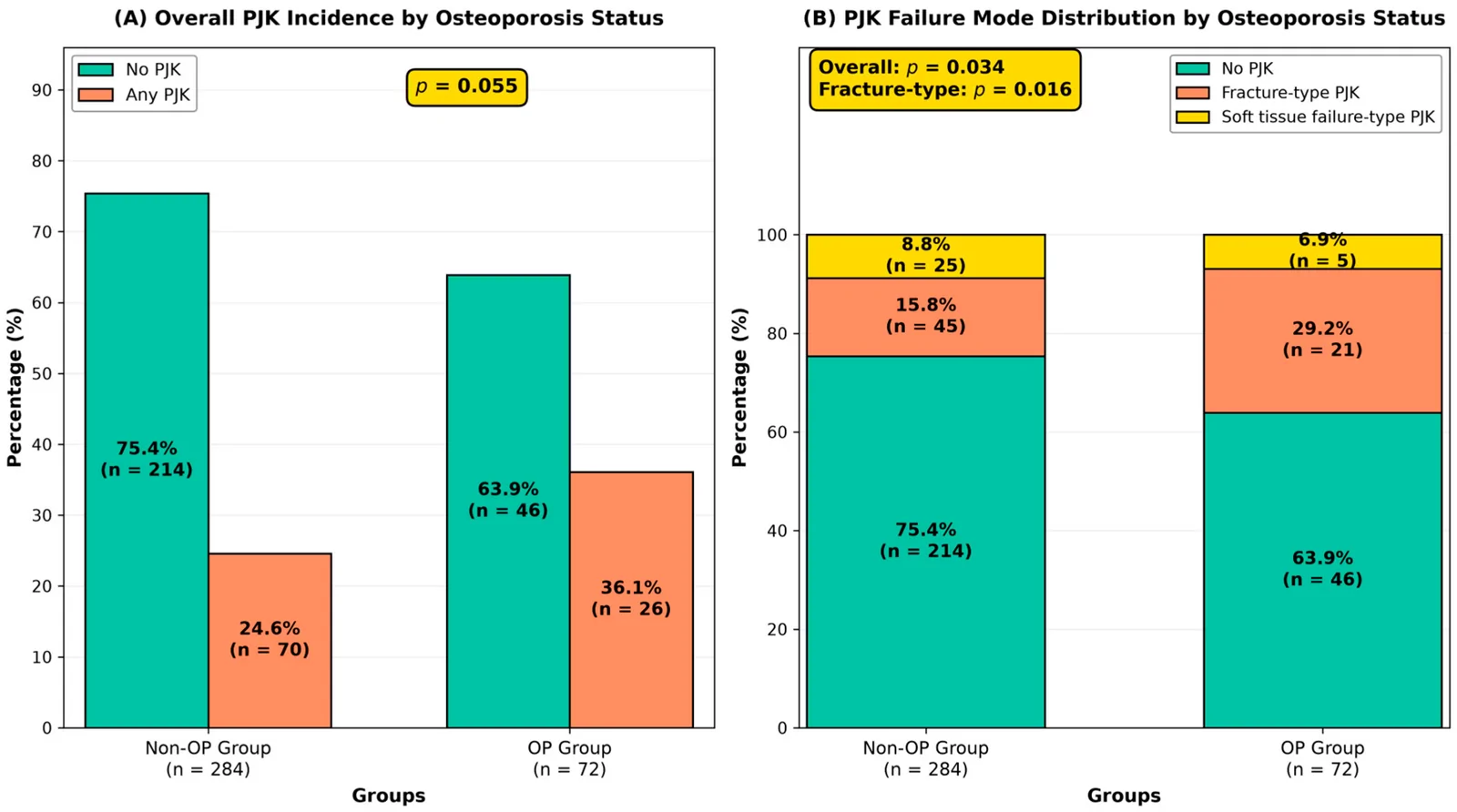
(**A**) Incidence of PJK and (**B**) distribution of PJK modes according to osteoporosis status.

The overall incidence of PJK showed a trend toward higher rates in the OP group (36.1%, 26/72) compared to the non-OP group (24.6%, 70/284), although this difference did not reach statistical significance (relative risk = 1.47, 95% CI = 0.99–2.18; absolute risk difference = 11.5%, 95% CI = −0.5% to 23.5%; *p* = 0.055) (Figure 1A). After adjustment for age, sex, previous lumbar fusion, ALIF at L5-S1, LLIF at ≥L4-5, pelvic incidence and preoperative PTH use, the association was attenuated and remained non-significant (adjusted OR = 1.60, 95% CI = 0.87–2.92, *p* = 0.129), whereas fracture-type PJK retained a borderline association (crude OR = 2.19, 95% CI = 1.20–3.98, *p* = 0.011; adjusted OR = 1.90, 95% CI = 0.98–3.67, *p* = 0.058). The distribution of PJK modes differed significantly between the groups (*p* = 0.034) (Figure 1B). Notably, fracture-type PJK occurred nearly twice as frequently in the OP group compared to the non-OP group (29.2% vs. 15.8%, *p* = 0.016). In contrast, the rates of soft tissue failure-type PJK were similar between the groups with 6.9% vs. 8.8%, respectively. Among patients who developed PJK, most events occurred within the first three months postoperatively in both groups (median: 2.35 months in the non-OP group and 2.95 months in the OP group). During the 2-year follow-up, 42 of 356 patients (11.8%) underwent revision surgery for junctional complications. Revision was more frequent after fracture-type than after soft tissue failure-type PJK (27 of 66, 40.9% vs. 6 of 30, 20.0%; *p* = 0.063). The remaining nine revisions occurred in patients who did not meet the radiographic definition of PJK (9 of 260, 3.5%). The overall revision rate did not differ between the two groups (11.6% vs. 12.5%, *p* = 0.839).

### 3.3. Univariate Analysis of Risk Factors for PJK

In the non-OP group, PJK was associated with older age (70.8 vs. 68.7 years, *p* = 0.024), higher ASA grade (*p* = 0.011), and lower HU at UIV (110.4 vs. 122.2, *p* = 0.035) (Table 2). No significant risk factors were observed in surgical variables (Table 3). Radiographic factors included higher preoperative PJA (2.2° vs. −1.8°, *p* < 0.001), lower postoperative PI-LL mismatch (2.6° vs. 7.5°, *p* = 0.002), higher age-adjusted PI-LL offset (8.3° vs. 3.3°, *p* < 0.001), and higher L1PA offset (−1.2° vs. −6.0°, *p* < 0.001) (Table 4). In the OP group, no demographic factors were associated with PJK development (Table 2). Regarding surgical variables, UIV levels differed significantly between patients with and without PJK: significantly more patients in the PJK group underwent thoracolumbar junction (T11-L1) stop compared to the non-PJK group (61.5% vs. 34.8%, *p* = 0.028) (Table 3). Analysis of radiographic factors showed that PJK was associated with higher preoperative PJA (1.1° vs. −3.5°, *p* = 0.003), lower postoperative PI-LL mismatch (6.4° vs. 13.4°, *p* = 0.018), and higher age-adjusted PI-LL offset (5.7° vs. −2.3°, *p* = 0.010) (Table 4). Intraclass correlation coefficients (two-way random effects, absolute agreement) were ICC = 0.942 (95% CI 0.884–0.974) for preoperative PJA, ICC = 0.928 (95% CI 0.851–0.964) for postoperative L1PA, ICC = 0.978 (95% CI 0.952–0.989) for postoperative PI-LL, and ICC = 0.977 (95% CI 0.949–0.989) for HU at UIV. Agreement on the fracture-type versus soft tissue failure-type classification was κ = 0.706 (95% CI 0.557–0.855).

### 3.4. Multivariate Analysis of Risk Factors for PJK in Each Osteoporosis Group

Multivariable logistic regression identified the variables independently associated with PJK within each group (Table 5). These are the variables that reached significance in each group and should not be read as evidence that the underlying effects differ between groups; formal interaction testing is reported in Supplementary Table S1. In the non-OP group, independent predictors included lower HU at UIV (odds ratio [OR] = 0.990, 95% CI = 0.982–0.998, *p* = 0.011), higher preoperative PJA (OR = 1.098, 95% CI = 1.050–1.149, *p* < 0.001), and higher L1PA offset (OR = 1.136, 95% CI = 1.070–1.207, *p* < 0.001). In the OP group, independent predictors included higher preoperative PJA (OR = 1.149, 95% CI = 1.039–1.270, *p* = 0.007) and higher age-adjusted PI-LL offset (OR = 1.052, 95% CI = 1.003–1.103, *p* = 0.039). When the same non-osteoporotic model was refitted with fracture-type PJK as the outcome, the association with HU at the UIV was considerably stronger (OR = 0.977, 95% CI = 0.966–0.989, *p* < 0.001) than against the combined endpoint, indicating that combining the two failure modes dilutes this association. The clinically prespecified model reproduced these results. The three variables identified in the non-osteoporotic group remained significant in the prespecified model with almost identical effect sizes, as did preoperative PJA and the age-adjusted PI-LL offset in the osteoporotic group (Supplementary Table S3). In a sensitivity analysis restricted to the 325 patients whose parameters were all measured on the 6-week radiograph, every predictor remained statistically significant and discrimination was unchanged (AUC 0.743 and 0.782; Supplementary Table S4).

### 3.5. Predictive Performance of Identified Risk Factors

**Figure 2:**
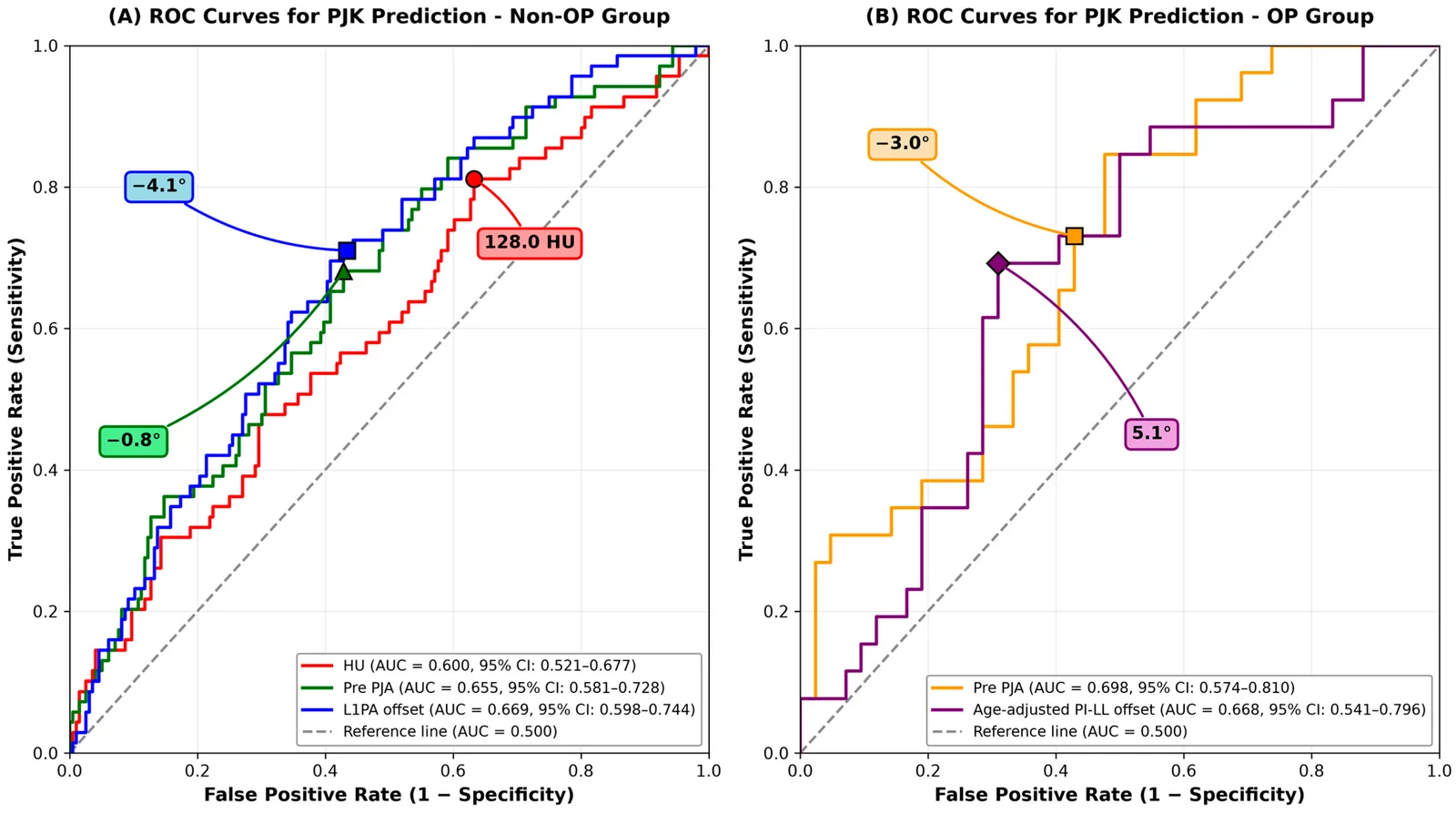
ROC curves for individual risk factors in predicting PJK for (**A**) the non-OP group and (**B**) the OP group.

**Figure 3:**
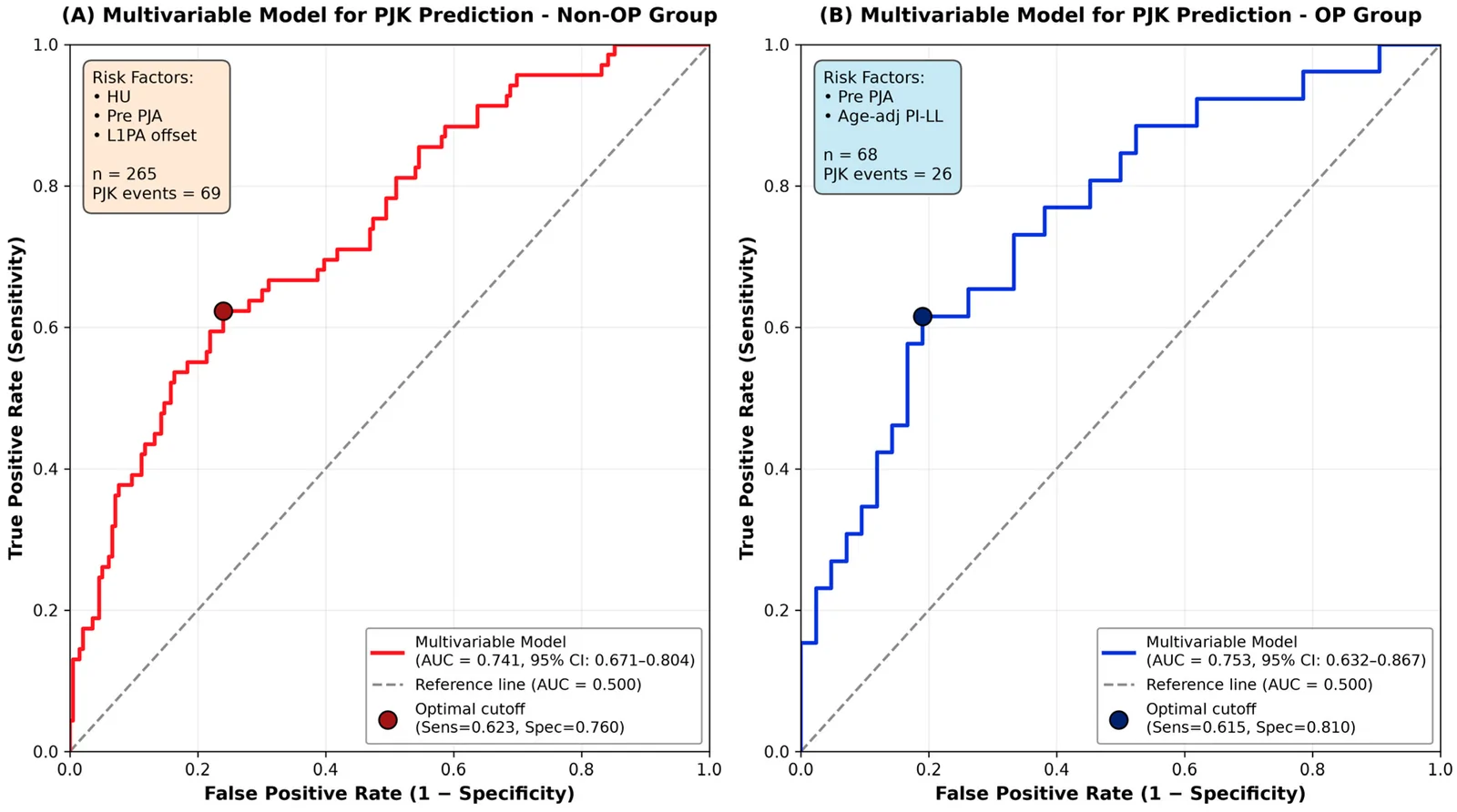
Performance of the multivariable model for predicting PJK in (**A**) the non-OP group and (**B**) the OP group.

In the non-OP group, HU (AUC = 0.600, cutoff = 128.0 HU), preoperative PJA (AUC = 0.655, cutoff = −0.8°), and postoperative L1PA offset (AUC = 0.669, cutoff = −4.1°) showed only weak discriminative ability (Figure 2A). Discrimination was similarly weak in the OP group for preoperative PJA (AUC = 0.698, cutoff = −3.0°) and age-adjusted PI-LL offset (AUC = 0.668, cutoff = 5.1°) (Figure 2B); no cut-off derived from a single variable is adequate to guide operative strategy on its own. The multivariable models displayed acceptable discriminative ability for both groups: AUC = 0.741 (95% CI = 0.671–0.804) for the non-OP group (Figure 3A) and AUC = 0.753 (95% CI = 0.632–0.867) for the OP group (Figure 3B), corresponding to optimism-corrected values of 0.729 and 0.735 after bootstrap internal validation (Supplementary Table S2; calibration curves in Supplementary Figure S1). All receiver operating characteristic analyses were performed on the complete-case sample of the multivariable model within each group (non-OP *n* = 265, 69 events; OP *n* = 68, 26 events). Detailed performance metrics for the identified risk factors in predicting PJK are presented in Table 6.

## 4. Discussion

This study demonstrates that risk factors for PJK development differ between patients with and without osteoporosis following long-segment fusion for adult spinal deformity. In non-osteoporotic patients, three independent risk factors emerged: lower HU at the UIV, greater preoperative PJA, and a greater L1PA offset, indicating relative overcorrection. Conversely, in osteoporotic patients, higher preoperative PJA and a greater age-adjusted PI-LL offset, also indicating relative overcorrection, emerged as the primary risk factors. These findings are consistent with bone quality influencing the biomechanical environment and compensatory mechanisms, and they raise the hypothesis that preventive strategies may need to differ between the two populations.

The relationship between osteoporosis and PJK has been extensively investigated, with most studies reporting osteoporosis as a significant risk factor for PJK development [5,6,7,8,9,10]. Our study corroborates previous findings, indicating a trend toward higher PJK incidence in the osteoporotic group (36.1% vs. 24.6%, *p* = 0.055). More importantly, we demonstrated that osteoporotic patients had a significantly higher rate of fracture-type PJK compared to non-osteoporotic patients (29.2% vs. 15.8%, *p* = 0.016), highlighting the distinct failure mechanism in compromised bone. However, our study diverges from prior research in that the variables associated with PJK differed between osteoporotic and non-osteoporotic populations. Formal interaction testing confirmed a differential effect only for the L1PA offset, so this observation raises the possibility that prevention strategies may need to differ between these groups but does not establish it.

Both groups identified higher preoperative PJA as an independent predictor for the development of PJK. This finding is intuitive given that PJK is defined by the absolute magnitude of postoperative PJA; thus, a higher baseline PJA inherently increases the likelihood of meeting diagnostic criteria. Our findings align with previous studies that report that higher preoperative PJA increases the risk of PJK [18,19]. In the present study, the optimal cutoff values of preoperative PJA were −0.8° and −3.0° (both representing lordotic angle) for the non-OP and OP groups, respectively. These results suggest that even a relatively small pre-existing junctional kyphosis can predispose patients to PJK, regardless of osteoporosis status. However, the OP group demonstrated a smaller PJA cutoff value (−3.0° vs. −0.8°). The AUCs of preoperative PJA did not differ significantly between the groups (0.698 vs. 0.655; difference 0.043, 95% CI = −0.101 to 0.188, *p* = 0.558, DeLong test). These findings raise the possibility that, in patients with osteoporosis, avoiding a kyphotic segment at the proximal junction when selecting the UIV may be worth considering, although our data cannot establish that doing so reduces the risk of PJK. Although not statistically significant in the multivariate model, it is noteworthy that, in the OP group, fusion more frequently terminated at the thoracolumbar junction (UIV at T11-L1) in patients who developed PJK (Table 3). The thoracolumbar junction—a transitional zone characterized by reduced rib support and increased mobility—is considered potentially unstable. Stopping fusion at the thoracolumbar junction has long been regarded as a level of concern [20,21,22,23]. Kim et al. demonstrated that a UIV positioned at the thoracolumbar junction was associated with higher PJK rates compared to an upper thoracic UIV, particularly in older patients with osteoporosis [23]. Park et al. also identified osteoporosis as the most significant risk factor for proximal junctional failure (PJF; OR = 4.459) in cases where fusion ended at the thoracolumbar junction [21]. Therefore, patients with osteoporosis or requiring substantial lordosis correction may be less suitable candidates for UIV placement at this transitional region.

In the present study, we identified HU as a predictor of PJK in the non-osteoporotic patient population (cutoff = 128.0, AUC = 0.600). This finding is remarkably consistent with the growing literature establishing HU thresholds for proximal junctional complications in ASD surgery. Our cutoff of 128.0 HU falls within the range of 120–130 HU reported by multiple independent studies for lower thoracic and lumbar UIV levels [10,24,25]. In contrast to the non-OP group, HU did not demonstrate significant predictive value for PJK in the OP group. The OP group had a significantly lower mean HU than the non-OP group (90.8 vs. 119.3; Table 1), and within the OP group, mean HU values remained below 100 HU in patients both with and without PJK (89.7 vs. 92.4; Table 2). One possible explanation for the lack of association between HU and PJK in the osteoporotic group is a floor effect, since most osteoporotic patients already have HU values below the previously established risk threshold of 120–130. We advance this only as a hypothesis: our data cannot establish that risk ceases to increase below a critical threshold, and the observation is equally compatible with limited power in a subgroup of 72 patients. In comparison, the non-OP group included patients with a wider spectrum of bone density from osteopenia to normal, which may explain why HU retained predictive value for PJK in that group.

Regarding the extent of lordosis correction, the non-OP group demonstrated that only L1PA offset was significant in the multivariate analysis, although PI-LL mismatch, age-adjusted PI-LL offset, and L1PA offset were significant in the univariate analysis. The L1PA differs from the conventional PI-based LL evaluation (either PI-LL or age-adjusted PI-LL) in that L1PA encompasses the alignment from the hip joint to the L1 vertebra. Although no comparative studies among those lordosis correction parameters exist, the L1PA may provide more comprehensive insights into LL correction, such as magnitude and orientation to the hip joint, yielding better predictive performance for PJK. Previous studies have demonstrated that lower L1PA is associated with an increased risk of PJK. Duvvuri et al. found that the L1PA was significantly lower in the PJK group than in the non-PJK group (7.5° vs. 10.4°, *p* = 0.001) [26]. However, L1PA should be interpreted concerning the individual PI values because L1PA is strongly associated with PI in a normative cohort [27]. We found that greater L1PA offset relative to the normative L1PA value (i.e., relative overcorrection of L1PA) significantly increased the risk of PJK, indicating that overcorrection in terms of L1PA should be avoided during surgery. In the OP group, the L1PA was not a significant predictor for PJK, but the age-adjusted PI-LL offset emerged as a significant predictor of PJK, with higher offset values (indicating overcorrection relative to age-adjusted targets) associated with increased risk. This finding aligns with mounting evidence that avoidance of age-adjusted PI-LL overcorrection is critical in mitigating PJK risk [28,29,30,31]. In osteoporotic patients, the reduced structural capacity of the UIV and adjacent vertebrae renders them particularly vulnerable to this increased mechanical loading, predisposing them to early junctional failure. Although patients’ ages may not be perfectly correlated with their bone quality, it is reasonable to infer that elderly patients are likely to experience age-related deterioration in the structural integrity of bone and soft tissues, increasing susceptibility to junctional mechanical failure [6,32]. The present study also found that patients were significantly older in the OP group than in the non-OP group (72.0 vs. 69.2 years, *p* = 0.002; Table 1). This is consistent with restraint in sagittal correction, targeting age-adjusted rather than absolute normative alignment goals, in osteoporotic patients.

This study has several important limitations that warrant consideration. First, we defined osteoporosis based on DEXA T-scores, a clinically accessible and widely validated method for assessing bone mineral density. While other metrics exist (CT-based HU, MRI-based vertebral bone quality score) for assessing local bone quality [10,33,34], DEXA offers the advantages of simplicity, reproducibility, and routine availability in clinical practice, facilitating widespread implementation of our findings. Multiple studies have demonstrated strong correlations between T-scores and alternative bone quality metrics [35,36], suggesting that DEXA-based classification captures essential bone quality characteristics relevant to surgical outcomes. Nevertheless, future investigations incorporating multimodal bone quality assessments may provide additional insights into the relationship between specific aspects of bone microarchitecture and PJK risk. Site-specific T-scores were not retained separately in our database; only the lowest value among the measured regions was available, so a sensitivity analysis restricted to hip-based T-scores was not possible. Second, the relatively small sample size in the osteoporotic group (N = 72) raises concerns about statistical power and potential overfitting of our multivariate model. However, several factors mitigate this concern. We employed stepwise regression with stringent inclusion criteria (*p* < 0.05 in univariate analysis) to minimize the number of variables entered into the final model, reducing overfitting risk. The osteoporotic group model retained only two predictor variables with 26 PJK events, but these were selected from 30 candidate variables, so the events-per-variable ratio of the retained model overstates the stability of the selection procedure. Additionally, the identified risk factors (preoperative PJA and age-adjusted PI-LL offset) demonstrated biological plausibility and consistency with existing biomechanical principles, supporting their validity beyond statistical associations alone. Nevertheless, external validation in independent cohorts would strengthen confidence in these findings and their generalizability. Third, our analysis included both fracture-type and soft tissue failure-type PJK in the primary outcome definition. While osteoporosis predominantly affects bone quality and might be expected to influence fracture-type PJK more specifically, we elected to include both PJK modes for several reasons. First, even soft tissue failure can be a clinically significant complication that can lead to revision surgery and functional deterioration, justifying its inclusion in comprehensive risk factor analysis [7,37]. Second, osteoporotic patients may experience concurrent degradation of ligamentous structures and paraspinal muscle quality beyond bone mineral density decline alone, potentially predisposing them to soft tissue failure as well as vertebral fractures [38]. Third, early subtle fractures may not be readily apparent on plain radiographs, potentially leading to misclassification of some fracture-type PJK cases as soft tissue failure-type, which could introduce bias if only one failure mode were analyzed. Future investigations using advanced imaging modalities such as CT or MRI for PJK characterization may provide more precise differentiation of failure mechanisms and their distinct risk factors in osteoporotic populations. Fourth, preoperative PJA is partly built into the definition of PJK, since the diagnosis requires an increase of ≥10° from the preoperative value in addition to a postoperative PJA ≥20°. Its association with PJK should therefore be regarded as partly definition-driven rather than as that of a conventional independent risk factor. Fifth, our analysis focused on radiographic parameters measured at standardized time points and did not incorporate dynamic or functional assessments that might provide additional predictive value. Emerging evidence suggests that muscle quality, spinal flexibility, and compensatory mechanisms may influence junctional complications, particularly in elderly patients with osteoporosis [39,40]. Incorporating such multidimensional assessments into future risk stratification models may further refine our understanding of PJK pathogenesis in different patient populations. Sixth, some information was not captured systematically in our database. Bone-health management other than preoperative PTH use, including antiresorptive medication, calcium and vitamin D supplementation, and previous fragility fractures, was unavailable for analysis, so residual confounding by these factors cannot be excluded. Patient-reported outcome measures and neurological status were likewise not recorded consistently across the study period, so we cannot say how often radiographic PJK was accompanied by symptoms or functional deterioration; proximal junctional failure was not defined as a separate outcome, and revision surgery for junctional complications is therefore the only clinical consequence we are able to report. Seventh, nine patients who underwent reoperation during follow-up for an indication unrelated to junctional complications were excluded, because the original construct had been revised and subsequent radiographs no longer reflected it. Excluding patients on the basis of an event occurring after the index operation conditions the cohort on a post-baseline outcome and may introduce selection bias; these patients represented 2.2% of those screened, and radiographic follow-up of the original construct was not available for them. Most importantly, surgical strategy in this cohort was not randomly assigned but was adapted by the operating surgeon to the patient’s bone quality: correction targets, UIV selection, and the use of prophylactic measures such as cement augmentation were all influenced by preoperative bone mineral density. Stratifying by osteoporosis status after this clinical filtering therefore compares two cohorts that have already been differentially treated, and constitutes confounding by indication. Consequently, the associations we report cannot distinguish whether the identified factors are genuine biological effect modifiers or reflections of differential surgical decision-making, and a non-significant association between a prophylactic technique and PJK cannot be interpreted as evidence that the technique is ineffective. Neither the surgeon identifier nor the date of surgery was retained in the analysis dataset, so adjustment for individual surgeon or for operative era was not possible, and alignment targets, implants and osteoporosis management are likely to have changed over the 2014–2023 study period. Our findings should be read as descriptive and hypothesis-generating; establishing that bone-quality-stratified surgical strategies reduce PJK would require a prospective design with protocol-defined treatment assignment.

## 5. Conclusions

This study identified differences in the variables associated with PJK between osteoporotic and non-osteoporotic patients undergoing long-segment fusion for ASD. These differences are exploratory and may inform hypotheses for bone quality-stratified surgical strategies. For osteoporotic patients, conservative correction targets relative to age-adjusted alignment may warrant particular attention. For non-osteoporotic patients, UIV bone quality, junctional kyphotic segments, and L1PA overcorrection may be relevant to surgical planning. Future prospective studies should validate these risk stratification models and assess whether implementing these bone quality-specific strategies can reduce the burden of this common and challenging complication.

## Figures and Tables

**Table 1:** Baseline characteristics.

Variables	Overall Cohort (N = 356)	Non-OP Group (N = 284)	OP Group (N = 72)	*p*
Age (years)	69.8 ± 6.7	69.2 ± 6.8	72.0 ± 5.7	**0.002**
Sex (female), *n* (%)	315 (88.5%)	245 (86.3%)	70 (97.2%)	**0.009**
Diagnosis				0.199
Flatback deformity, *n* (%)	224 (62.9%)	174 (61.3%)	50 (69.4%)	
Kyphoscoliosis, *n* (%)	132 (37.1%)	110 (38.7%)	22 (30.6%)	
ASA grade				0.443
Grade 1, *n* (%)	18 (5.1%)	15 (5.3%)	3 (4.2%)	
Grade 2, *n* (%)	277 (77.8%)	217 (76.4%)	60 (83.3%)	
Grade 3, *n* (%)	61 (17.1%)	52 (18.3%)	9 (12.5%)	
m-FI5				0.612
Robust, *n* (%)	63 (17.7%)	50 (17.6%)	13 (18.1%)	
Pre-frail, *n* (%)	141 (39.6%)	116 (40.8%)	25 (34.7%)	
Frail, *n* (%)	152 (42.7%)	118 (41.5%)	34 (47.2%)	
BMI (kg/m^2^)	25.9 ± 3.9	26.1 ± 4.0	25.1 ± 3.3	0.056
T-score	−1.5 ± 1.4	−1.0 ± 1.2	−3.2 ± 0.7	**<0.001**
HU at UIV	113.7 ± 41.7	119.3 ± 41.0	90.8 ± 36.7	**<0.001**
Preoperative use of PTH, *n* (%)	43 (12.1%)	28 (9.9%)	15 (20.8%)	**0.011**
Prior lumbar fusion, *n* (%)	152 (42.7%)	129 (45.4%)	23 (31.9%)	**0.039**
ALIF at L5-S1, *n* (%)	154 (43.3%)	131 (46.1%)	23 (31.9%)	**0.030**
LLIF at ≥ L4-5, *n* (%)	239 (67.1%)	200 (70.4%)	39 (54.2%)	**0.009**
PSO, *n* (%)	36 (10.1%)	27 (9.5%)	9 (12.5%)	0.452
Cementing at UIV, *n* (%)	105 (29.5%)	80 (28.2%)	25 (34.7%)	0.276
TP hook at UIV+1, *n* (%)	76 (21.3%)	64 (22.5%)	12 (16.7%)	0.278
UIV screw angle (°)	2.9 ± 4.6	3.0 ± 4.6	2.6 ± 4.6	0.469
Number of rods				0.506
2 rods, *n* (%)	304 (85.4%)	245 (86.3%)	59 (81.9%)	
3 rods, *n* (%)	32 (9.0%)	25 (8.8%)	7 (9.7%)	
4 rods, *n* (%)	20 (5.6%)	14 (4.9%)	6 (8.3%)	
Fusion length (levels)	7.3 ± 1.9	7.3 ± 1.9	7.3 ± 1.8	0.981
Preoperative PI (°)	53.7 ± 10.8	53.0 ± 10.9	56.4 ± 10.0	**0.017**
Preoperative PI-LL (°)	40.9 ± 19.8	40.7 ± 20.0	42.0 ± 19.4	0.619
Preoperative SS (°)	20.9 ± 11.2	20.6 ± 11.3	21.8 ± 10.9	0.425
Preoperative PT (°)	32.8 ± 11.0	32.3 ± 11.1	34.5 ± 10.7	0.138
Preoperative TK (°)	9.2 ± 16.8	8.2 ± 16.8	13.0 ± 16.1	0.079
Preoperative T1PA (°)	32.9 ± 12.0	32.6 ± 12.3	34.0 ± 10.6	0.391
Preoperative SVA (mm)	79.9 ± 57.0	80.4 ± 56.8	77.9 ± 58.1	0.740
Preoperative PJA (°)	−1.0 ± 7.0	−0.8 ± 7.2	−1.8 ± 6.4	0.310

Bold *p* values indicate statistical significance.

**Table 2:** Univariate analysis of demographic data for each osteoporosis group.

Variables	Non-OP (N = 284)			OP (N = 72)		
	No PJK (N = 214)	PJK (N = 70)	*p*	No PJK (N = 46)	PJK (N = 26)	*p*
Age (years)	68.7 ± 6.4	70.8 ± 7.7	**0.024**	71.3 ± 6.4	73.2 ± 4.1	0.185
Sex (female), *n* (%)	183 (85.5%)	62 (88.6%)	0.519	44 (95.7%)	26 (100.0%)	0.281
Diagnosis			0.753			0.615
Flatback deformity, *n* (%)	130 (60.7%)	44 (62.9%)		31 (67.4%)	19 (73.1%)	
Kyphoscoliosis, *n* (%)	84 (39.3%)	26 (37.1%)		15 (32.6%)	7 (26.9%)	
ASA grade			**0.011**			0.641
Grade 1, *n* (%)	15 (7.0%)	0 (0.0%)		2 (4.3%)	1 (3.8%)	
Grade 2, *n* (%)	166 (77.6%)	51 (72.9%)		37 (80.4%)	23 (88.5%)	
Grade 3, *n* (%)	33 (15.4%)	19 (27.1%)		7 (15.2%)	2 (7.7%)	
m-FI5			0.946			0.869
Robust, *n* (%)	37 (17.3%)	13 (18.6%)		8 (17.4%)	5 (19.2%)	
Pre-frail, *n* (%)	87 (40.7%)	29 (41.4%)		17 (37.0%)	8 (30.8%)	
Frail, *n* (%)	90 (42.1%)	28 (40.0%)		21 (45.7%)	13 (50.0%)	
BMI (kg/m^2^)	26.1 ± 4.3	26.3 ± 3.2	0.651	24.6 ± 3.5	26.1 ± 2.8	0.074
T-score	−1.0 ± 1.3	−1.3 ± 1.0	0.053	−3.2 ± 0.7	−3.2 ± 0.8	0.942
HU at UIV	122.2 ± 40.0	110.4 ± 43.0	**0.035**	89.7 ± 37.3	92.4 ± 36.3	0.779
Preoperative use of PTH, *n* (%)	19 (8.9%)	9 (12.9%)	0.332	9 (19.6%)	6 (23.1%)	0.725

Bold *p* values indicate statistical significance. HU at UIV was available in 273 of 284 non-OP and 66 of 72 OP patients.

**Table 3:** Univariate analysis of surgical variables for each osteoporosis group.

Variables	Non-OP (N = 284)			OP (N = 72)		
	No PJK (N = 214)	PJK (N = 70)	*p*	No PJK (N = 46)	PJK (N = 26)	*p*
Prior lumbar fusion, *n* (%)	98 (45.8%)	31 (44.3%)	0.826	17 (37.0%)	6 (23.1%)	0.225
ALIF at L5-S1, *n* (%)	92 (43.0%)	39 (55.7%)	0.064	14 (30.4%)	9 (34.6%)	0.715
LLIF at ≥L4-5, *n* (%)	146 (68.2%)	54 (77.1%)	0.156	22 (47.8%)	17 (65.4%)	0.151
PSO, *n* (%)	23 (10.7%)	4 (5.7%)	0.213	6 (13.0%)	3 (11.5%)	0.853
Cementing at UIV, *n* (%)	61 (28.5%)	19 (27.1%)	0.826	14 (30.4%)	11 (42.3%)	0.309
TP hook at UIV+1, *n* (%)	48 (22.4%)	16 (22.9%)	0.941	8 (17.4%)	4 (15.4%)	0.826
UIV screw angle (°)	3.1 ± 4.8	2.7 ± 4.1	0.531	2.1 ± 4.1	3.4 ± 5.5	0.257
Number of rods			0.539			0.505
2 rods, *n* (%)	182 (85.0%)	63 (90.0%)		36 (78.3%)	23 (88.5%)	
3 rods, *n* (%)	21 (9.8%)	4 (5.7%)		5 (10.9%)	2 (7.7%)	
4 rods, *n* (%)	11 (5.1%)	3 (4.3%)		5 (10.9%)	1 (3.8%)	
Fusion length (levels)	7.4 ± 2.0	7.0 ± 1.6	0.178	7.5 ± 1.8	7.0 ± 1.6	0.198
UIV levels			0.064			**0.028**
T11-L1, *n* (%)	89 (41.6%)	38 (54.3%)		16 (34.8%)	16 (61.5%)	
≥T10, *n* (%)	125 (58.4%)	32 (45.7%)		30 (65.2%)	10 (38.5%)	

Bold *p* values indicate statistical significance. The UIV screw angle was available in 273 of 284 non-OP and 71 of 72 OP patients.

**Table 4:** Univariate analysis of radiographic data in each osteoporosis group.

Variables	Non-OP (N = 284)			OP (N = 72)		
	No PJK (N = 214)	PJK (N = 70)	*p*	No PJK (N = 46)	PJK (N = 26)	*p*
Preoperatively						
PI (°)	52.8 ± 10.9	53.3 ± 10.8	0.747	57.4 ± 10.1	54.5 ± 9.7	0.232
PI-LL (°)	41.2 ± 20.2	39.1 ± 18.8	0.440	42.3 ± 19.2	41.4 ± 20.0	0.858
SS (°)	20.9 ± 11.0	19.9 ± 12.2	0.504	23.9 ± 9.5	20.1 ± 12.3	0.102
PT (°)	32.0 ± 11.2	33.3 ± 10.8	0.396	33.5 ± 9.9	34.3 ± 12.0	0.585
TK (°)	9.2 ± 16.8	5.2 ± 16.6	0.163	15.0 ± 16.3	8.7 ± 15.4	0.201
T1PA (°)	32.4 ± 12.5	33.3 ± 11.7	0.601	33.7 ± 10.6	34.5 ± 10.8	0.747
SVA (mm)	81.1 ± 57.6	78.5 ± 54.8	0.740	79.9 ± 63.7	74.4 ± 47.6	0.701
PJA (°)	−1.8 ± 6.8	2.2 ± 7.4	**<0.001**	−3.5 ± 5.8	1.1 ± 6.3	**0.003**
At 6 weeks postoperatively						
Change in LL (°)	33.9 ± 21.4	36.4 ± 20.7	0.395	29.0 ± 20.7	34.6 ± 19.0	0.263
PI-LL mismatch (°)	7.5 ± 12.0	2.6 ± 9.9	**0.002**	13.4 ± 12.3	6.4 ± 10.8	**0.018**
Age-adjusted PI-LL offset	3.3 ± 12.9	8.3 ± 10.1	**<0.001**	−2.3 ± 12.8	5.7 ± 11.4	**0.010**
L1PA offset (°)	−6.0 ± 5.6	−1.2 ± 4.6	**<0.001**	−5.4 ± 5.8	−5.1 ± 5.5	0.826

Bold *p* values indicate statistical significance. Preoperative PJA was available in 276 of 284 non-OP and 68 of 72 OP patients, and preoperative thoracic kyphosis in 189 of 284 and 49 of 72.

**Table 5:** Stepwise multivariable logistic regression analyses of factors associated with PJK within each osteoporosis stratum.

For Non-OP Group (*n* = 265, 69 PJK Events)			
Risk factors	Odds ratio	95% CI	*p*
HU at UIV	0.990	0.982–0.998	0.011
Preoperative PJA	1.098	1.050–1.149	<0.001
L1PA offset	1.136	1.070–1.207	<0.001
For OP group (*n* = 68, 26 PJK events)			
Risk factors	Odds ratio	95% CI	*p*
Preoperative PJA	1.149	1.039–1.270	0.007
Age-adjusted PI-LL offset	1.052	1.003–1.103	0.039

**Table 6:** Performance ability to predict PJK in each osteoporosis group.

For non-OP group (*n* = 265, 69 PJK events)							
Risk factor	Cutoff	AUC	Sensitivity	Specificity	PPV	NPV	Accuracy
Hounsfield units	128.0	0.600	81.2%	36.7%	31.1%	84.7%	48.3%
Preoperative PJA	−0.8°	0.655	68.1%	57.1%	35.9%	83.6%	60.0%
L1PA offset	−4.1°	0.669	71.0%	56.6%	36.6%	84.7%	60.4%
Multivariable model	NA	0.741	62.3%	76.0%	47.8%	85.1%	72.5%
For OP group (*n* = 68, 26 PJK events)							
Risk factor	Cutoff	AUC	Sensitivity	Specificity	PPV	NPV	Accuracy
Preoperative PJA	−3.0°	0.698	73.1%	57.1%	51.4%	77.4%	63.2%
Age-adjusted PI-LL offset	5.1°	0.668	69.2%	69.0%	58.1%	78.4%	69.1%
Multivariable model	NA	0.753	61.5%	81.0%	66.7%	77.3%	73.5%

## Data Availability

The data presented in this study are available on request from the corresponding author due to privacy and ethical restrictions.

## Supplementary Materials

The following supporting information can be downloaded at: https://www.mdpi.com/article/10.3390/jcm15166479/s1, Figure S1: Calibration of the multivariable models in the non-osteoporotic (A) and osteoporotic (B) groups; Figure S2: Patient flow; Table S1: Formal tests of interaction between osteoporosis status and each candidate predictor of proximal junctional kyphosis, fitted in the whole cohort (N = 356); Table S2: Bootstrap internal validation of the multivariable models (1000 resamples, Harrell’s optimism-correction procedure); Table S3: Clinically prespecified multivariable model of PJK within each osteoporosis stratum; Table S4: Sensitivity analysis excluding patients whose PJK was identified before the 6-week visit (N = 325).

## Abbreviations

The following abbreviations are used in this manuscript:

ALIF	Anterior lumbar interbody fusion
ASA	American Society of Anesthesiologists
ASD	Adult spinal deformity
AUC	Area under the curve
BMI	Body mass index
CI	Confidence interval
DEXA	Dual-energy X-ray absorptiometry
HU	Hounsfield unit
L1PA	L1 pelvic angle
LL	Lumbar lordosis
LLIF	Lateral lumbar interbody fusion
mFI-5	Modified frailty index-5
NPV	Negative predictive value
OP	Osteoporosis
PI	Pelvic incidence
PI-LL	Pelvic incidence minus lumbar lordosis
PJA	Proximal junctional angle
PJF	Proximal junctional failure
PJK	Proximal junctional kyphosis
PPV	Positive predictive value
PSO	Pedicle subtraction osteotomy
PT	Pelvic tilt
PTH	Parathyroid hormone
SS	Sacral slope
SVA	Sagittal vertical axis
T1PA	T1 pelvic angle
TK	Thoracic kyphosis
TP	Transverse process
UIV	Upper instrumented vertebra
