# Supplementary material for: Risk Factors for Proximal Junctional Kyphosis in Osteoporotic Versus Non-Osteoporotic Patients After Adult Spinal Deformity Surgery
Source: J Clin Med. 2026 Aug 21;15(16):6479. doi: 10.3390/jcm15166479 (PMC13514197; doi:10.3390/jcm15166479)

Supplementary Materials

Risk Factors for Proximal Junctional Kyphosis in Osteoporotic Versus Non-Osteoporotic Patients After Adult Spinal Deformity Surgery

Tae Soo Shin, Jin-Sung Park, Dong-Ho Kang, Jun-Seok Oh and Se-Jun Park *

* Correspondence

Table S1. Formal tests of interaction between osteoporosis status and each candidate predictor of proximal junctional kyphosis, fitted in the whole cohort (N = 356).

**Predictor**	**OR (non-OP)**	**OR (OP)**	**Ratio of ORs (interaction)**	**95% CI**	***p* for interaction**
Age	1.053	1.064	1.011	0.913–1.120	0.833
HU at UIV	0.992	1.002	1.010	0.994–1.025	0.213
Preoperative PJA	1.085	1.132	1.044	0.945–1.152	0.397
Postoperative PI-LL mismatch	0.962	0.948	0.986	0.936–1.039	0.596
Age-adjusted PI-LL offset	1.043	1.057	1.014	0.964–1.067	0.593
L1PA offset	1.132	1.010	0.892	0.805–0.988	0.029
ASA grade	2.329	0.636	0.273	0.069–1.081	0.064
UIV at T11-L1 (vs ≥T10)	1.668	3.000	1.799	0.578–5.594	0.311

Table S2. Bootstrap internal validation of the multivariable models (1000 resamples, Harrell's optimism-correction procedure).

**Internal validation (1000 bootstrap resamples)**	**non-OP model**	**OP model**
Apparent AUC	0.741	0.753
Optimism	0.012	0.018
Optimism-corrected AUC	0.729	0.735
Calibration slope (corrected)	0.947	0.949
Calibration-in-the-large	0.000	0.000
Brier score (corrected)	0.168	0.211
Brier score, null model	0.193	0.236

The calibration slope reported here is the bootstrap optimism-corrected value. The slopes shown in Figure S1 (0.87 and 0.71) are cross-validated estimates obtained by a different resampling procedure.

Table S3. Clinically prespecified multivariable model of PJK within each osteoporosis stratum.

**Variables**	**OR (non-OP)**	***p***	**OR (OP)**	***p***
Age	0.996	0.886	0.996	0.957
Body mass index	1.038	0.357	1.160	0.118
HU at UIV	0.990	0.014	1.001	0.954
Previous lumbar fusion	0.892	0.734	0.669	0.610
UIV at T11-L1	1.229	0.524	2.816	0.118
Pelvic fixation	1.030	0.944	2.022	0.453
Cement augmentation at UIV	1.052	0.894	3.208	0.125
TP hook at UIV+1	1.134	0.759	1.054	0.961
Preoperative PJA	1.091	<0.001	1.188	0.010
Age-adjusted PI-LL offset	1.021	0.222	1.012	0.043
L1PA offset	1.106	0.004	0.973	0.656

Table S4. Sensitivity analysis excluding patients whose PJK was identified before the 6-week visit (N = 325).

**Risk factor**	**OR**	**95% CI**	***p***
**non-OP group (n = 241, 45 events; AUC = 0.743)**			
HU at UIV	0.988	0.979–0.998	0.016
Preoperative PJA	1.102	1.045–1.162	<0.001
L1PA offset	1.138	1.061–1.220	<0.001
**OP group (n = 62, 20 events; AUC = 0.782)**			
Preoperative PJA	1.131	1.028–1.245	0.012
Age-adjusted PI-LL offset	1.057	1.004–1.113	0.036

Figure S1. Calibration of the multivariable models in the non-osteoporotic (A) and osteoporotic (B) groups. Apparent and cross-validated calibration curves are plotted against the line of ideal calibration, together with the observed proportion of PJK within equal-sized strata of predicted risk (five in the non-osteoporotic and four in the osteoporotic group) and a histogram of the predicted probabilities along the horizontal axis. The cross-validated calibration slopes were 0.87 and 0.71.

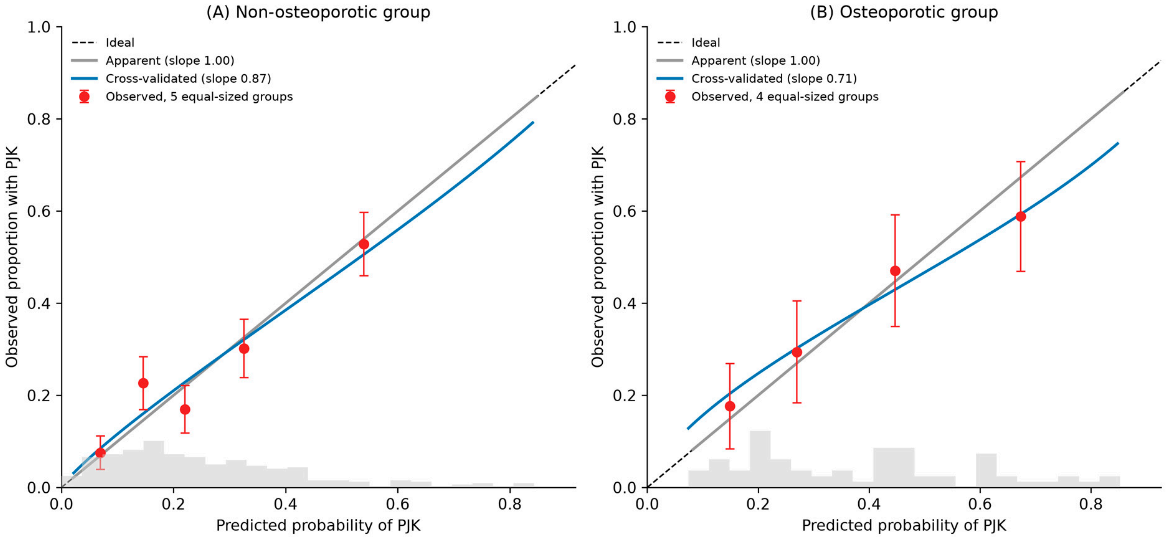

Figure S2. Patient flow.

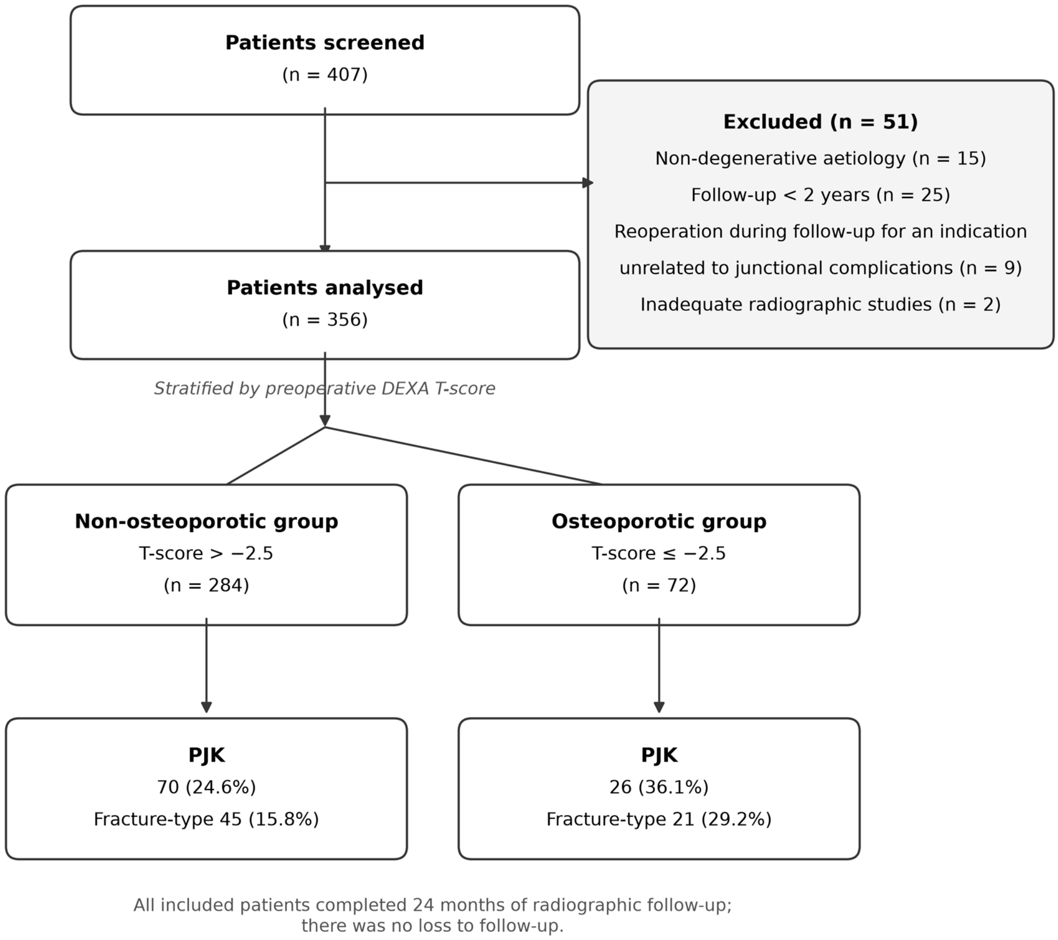

Supplement: Supplementary file 1 [file jcm-15-06479-s001.zip › jcm-4469704-supplementary.pdf]
